# Supplementary material for: Schottky barrier lowering due to interface states in 2D heterophase devices
Source: Nanoscale Adv. 2020 Dec 7;3(2):567–74. doi: 10.1039/d0na00795a (PMC9418679; doi:10.1039/d0na00795a)
Supplement: NA-003-D0NA00795A-s001 [file NA-003-D0NA00795A-s001.pdf]

Electronic Supplementary Material (ESI) for Nanoscale Advances.
This journal is © The Royal Society of Chemistry 2020

Schottky barrier lowering due to interface states in 2D heterophase devices
Supplementary Information

Line Jelver*[1,2], Daniele Stradi[1], Kurt Stokbro[1], and Karsten Wedel Jacobsen[2]

[1]Synopsys Denmark, Fruebjergvej 3, PostBox 4, DK-2100 Copenhagen, Denmark.
[2]Center for Atomic-scale Materials Design (CAMD), Technical University of Denmark, Bldg. 309, DK-2800 Lyngby, Denmark.

July 24, 2020

All calculations have been performed with QuantumATK 2019.03-SP1.[1]

A Computational details

The setup and relaxation of the device follows four steps which are illustrated in Figure 1.

1. The 1H and 1T' phases are set up in the conventional unit cell and relaxed using periodic boundary conditions. This gives the 2D lattice parameters $a = 3.59$ Å for the 1H phase and $a = 3.52$ Å, $b = 6.37$ Å for the 1T' phase which compares well with the experimental values.[2,3]
2. The interface is created using the interface builder in QuantumATK.[4,5] The 1T' phase is strained by 2.12 % along the y-direction to make a match between the (200)-edge of 1T' and the (02-20)-edge of 1H. We choose to strain the 1T' phase in order to maintain the semiconductor properties.
3. A nanoribbon of this interface is created with 18 atomic layers of each phase and 20 Å of vacuum between periodic images in the x-direction. The 6 atomic layers closest to the interface are allowed to relax and the remaining 1T' phase is fixed while the remaining 1H phase is kept rigid to allow for a compression or elongation of the interface region.
4. The relaxed interface is converted to a device configuration in order to perform the NEGF calculations. The central region is composed of about 6 nm's of 1T' phase and 19 nm's of 1H phase. For these calculation we use Dirichlet boundary conditions between the central region and electrodes, periodic boundary conditions in the y-direction and Neumann boundary conditions in the z-direction to avoid electrostatic interactions between neighbouring interface dipoles. The cell height is 15 nm's which ensures that the out-of-plane fields due to the 2D interface are properly accounted for and that their effect on the size of the barrier is minimized.[6,7,8]

*jelver@synopsys.com

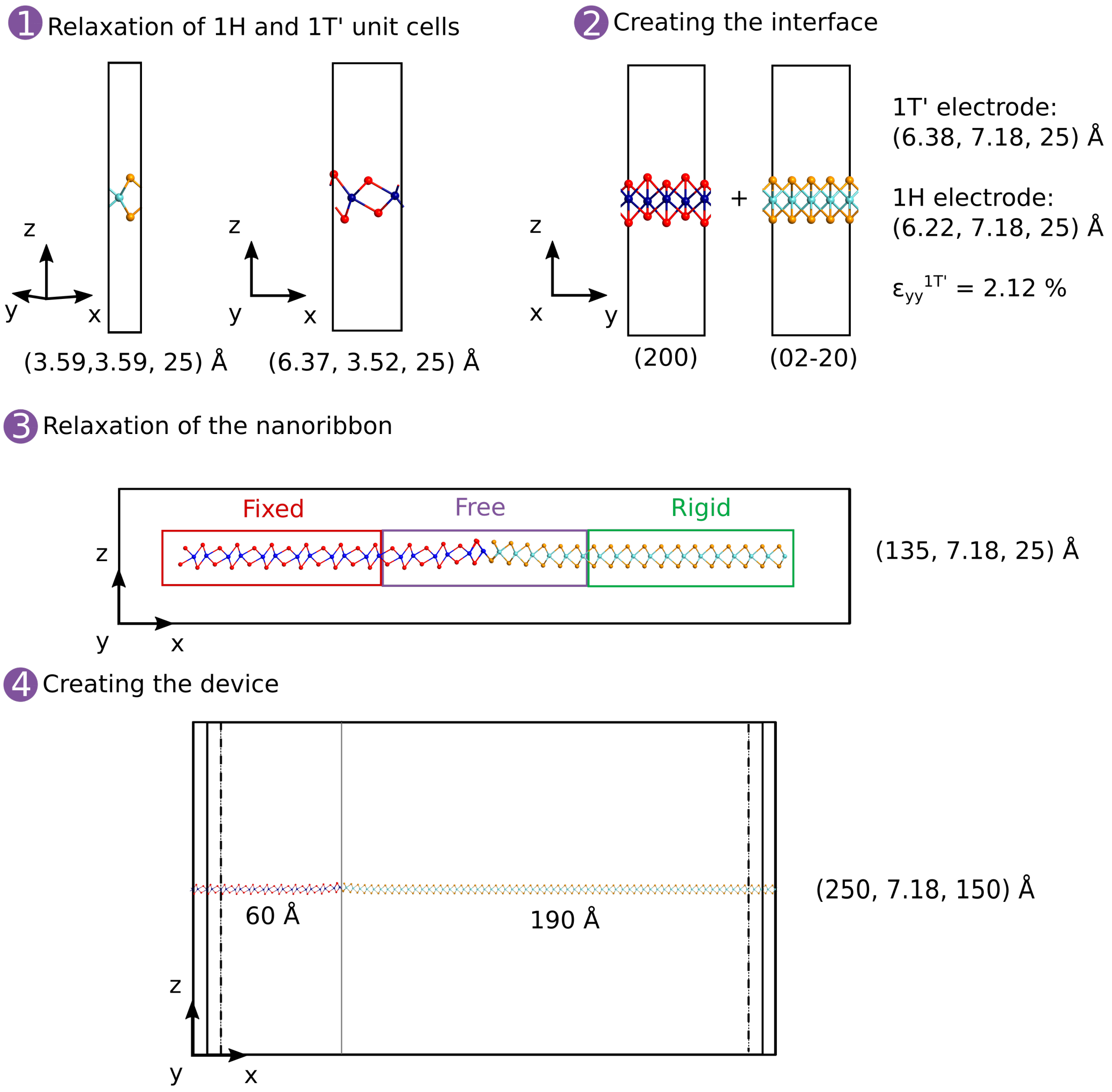

Figure 1: The relaxation scheme for setting up the device along with the unit cells of each calculation.

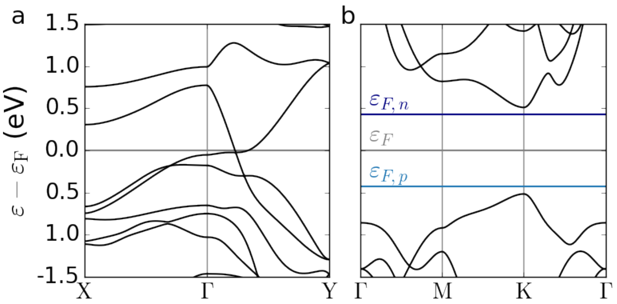

Figure 2: Bandstructure of the monolayer **a** 1T' phase and **b** 1H phase of $MoTe_2$. $\varepsilon_{F,n}$ and $\varepsilon_{F,p}$ show the Fermi level of the 1H phase with n- and p-doping concentrations of $N_{D/A} = 4.9 \times 10^{11}$ cm^{-2}.

All the structure relaxations use a force tolerance of 0.02 eV/Å and the k-point grid for the isolated 1H phase is (7, 7, 1) while it is (5, 11, 1) for the isolated 1T' phase and (k_x, 6, 1) for the remaining calculations. $k_x = 1$ for the nanoribbon calculation and $k_x = 401$ for the NEGF calculation. The occupations are described by using a Fermi-Dirac occupation function with an electronic temperature of 300 K.

B Bandstructures

The band diagrams of the 1T' and 1H phase of $MoTe_2$ can be seen in Figure 2 including the relative placement of the Fermi level for a n- or p-doping of $N_{D/A} = 4.9 \times 10^{11}$ cm^{-2}.

C Substrate effect

The depletion width scales with the effective dielectric constant of the 2D material which is modified by the surrounding dielectric subtrate.[9,10] We therefore investigate the effect of scaling the depletion width.

Using the Wentzel–Kramers–Brillouin (WKB) approximation and atomic units, the tunneling through an exponential barrier placed at $x = 0$ with height Φ^{SB} and depletion width x_D is given by,

$$T(E) = \exp\left(-2\int_0^{x_{cl}} \sqrt{2(V(x) - E)}\,\mathrm{d}x\right)$$
$$V(x) = \Phi^{SB}\mathrm{e}^{-x/x_D},\ x > 0$$

Where x_{cl} is the classical turning point of the barrier. We make the substitution $z = \mathrm{e}^{-\mathrm{x}/\mathrm{x}_\mathrm{D}}$ resulting in,

$$T(E) = \exp\left(-\sqrt{2\Phi^{SB}}\,x_D \int_{E/\Phi^{SB}}^{1} \frac{\sqrt{z - E/\Phi^{SB}}}{z}\,\mathrm{dz}\right).$$

Scaling the depletion width by s, $x_D \rightarrow s\,x_D$, will therefore result in a transmission,

$$T(E) \rightarrow (T(E))^s$$

scaling	$T_{ITE>ITUN}$
0.5 x_D	1765 K
1.0 x_D	743 K
1.5 x_D	340 K
2.0 x_D	250 K

Table 1: Effect of scaling the depletion width of the n-doped device, $N_D = 4.6 \times 10^{12}$ cm^{-2}.

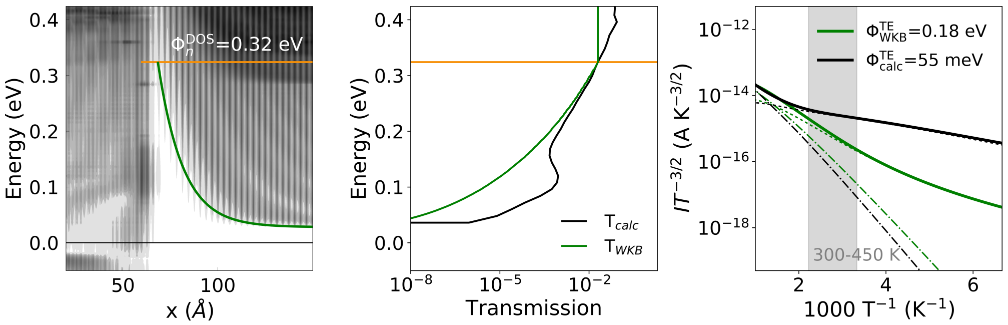

Figure 3: Conduction band bending, transmission spectrum, and Arrhenius plot of the device with a doping of $N_D = 4.6 \times 10^{12}$ cm^{-2} compared to a WKB model. **left** show the fitted band bending (green) and DOS barrier (orange). **middle** show the transmission spectrum of the calculation and the WKB model respectively. **right** show the Arrhenius plots and TE barriers resulting from the two transmission spectra shown in the middle part.

We have scaled the tunneling part of the transmission of the n-doped device with $N_D = 4.6 \times 10^{12}$ cm^{-2} using $s = 0.5$, $s = 1.5$ and $s = 2$ to investigate the effect of including a substrate. This results in a shift of the two regimes in Figure 4g in the main text. In Table 1, we list the temperature at which the thermionic current becomes larger than the tunneling current, $T_{ITE>ITUN}$. This shows that the tunneling current remains non-negligible for a depletion width up to twice the size of the calculated width.

D WKB model to evaluate the effect of the interface states.

The conduction band bending of the device with doping $N_D = 4.6 \times 10^{12}$ cm^{-2} is fitted to an exponential function which yields,

$$CB(x) = 55.3 \, \mathrm{e}^{-x/13.1},$$

when the unit of energy is eV and the unit of x is Angstrom. The fit can be seen on Figure 3. From this potential barrier, we can calculate the corresponding transmission using the WKB approximation as explained in section C. This transmission is seen together with the result of the calculation in the middle part of Figure 3. We have scaled the WKB transmission to reach the same value as the calculated transmission at the top of the barrier. Above the barrier, we set the WKB transmission to be constant at this value. This is a way of including that the availability of states for transport limits the transmission. As it can be seen on the figure, the WKB model show a very similar transmission except that the peak caused by the interface states is absent.

The current is now calculated using equation (2) in the main text and an Arrhenius plot is used to find the TE barrier. The result can be seen in the right part of figure 3 and show a large

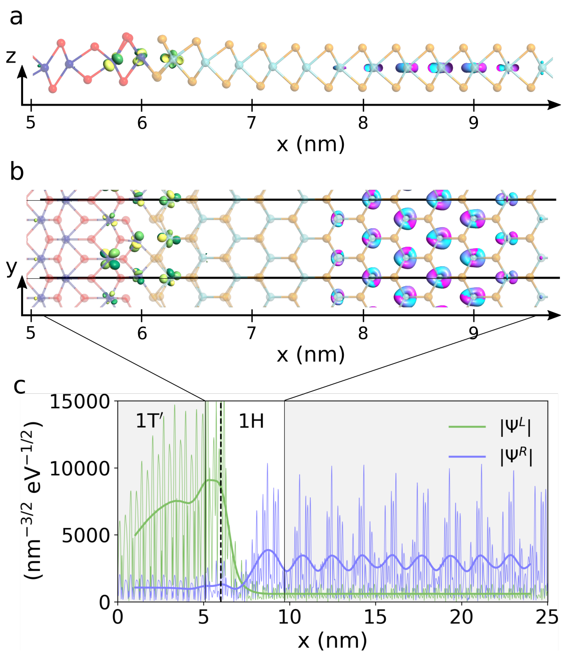

Figure 4: Transmission eigenstates of the device with p-doping $N_A = 4.6 \times 10^{12}$ cm^{-2} at $\varepsilon = -0.16$ eV and $k_y = -0.27$. **a** and **b** show the isosurfaces of the eigenstate from the 1T electrode, Ψ^L, (green and yellow isosurface) and the eigenstate from the 1H electrode, Ψ^R, (cyan and pink isosurface) seen from the side and top of the ML respectively. **c** shows the norm of the two eigenstates summed over the yz-plane and projected along the x-axis. The fat trend lines have been created using Gaussian smoothing.

difference between the tunneling current from the calculation and the tunneling current from the WKB model. This results in very different TE barrier heights between 300 and 450 K; 55 meV from the calculation and 0.18 eV from the WKB model. We believe this strongly supports our claim, that it is the interface states which are responsible for the very low barrier. Note, that the thermionic current of the two models are almost identical between 300 and 450 K which justifies that we have chosen the transmission of the WKB model to be constant above the barrier.

E Transmission eigenstates of the highly p-doped device.

The transmission eigenstates of the p-doped device at 0.16 eV below the Fermi level and at the k_y-value of -0.27, indicated by the white star on Figure 4f in the main text, are shown on Figure 4a and 4b. Figure 4c show the norm of the two transmission eigenstates. The behavior is generally the same as observed in the n-doped device.

References

[1] Søren Smidstrup, Troels Markussen, Pieter Vancraeyveld, Jess Wellendorff, Julian Schneider, Tue Gunst, Brecht Verstichel, Daniele Stradi, Petr A Khomyakov, Ulrik G Vej-Hansen, Maeng-Eun Lee, Samuel T Chill, Filip Rasmussen, Gabriele Penazzi, Fabiano Corsetti, Ari Ojanperä, Kristian Jensen, Mattias L N Palsgaard, Umberto Martinez, Anders Blom, Mads Brandbyge, and Kurt Stokbro. QuantumATK: an integrated platform of electronic and atomic-scale modelling tools. *Journal of Physics: Condensed Matter*, 32(1):015901, oct 2019. doi: 10.1088/1361-648x/ab4007.

[2] Ying Wang, Jun Xiao, Hanyu Zhu, Yao Li, Yousif Alsaid, King Yan Fong, Yao Zhou, Siqi Wang, Wu Shi, Yuan Wang, Alex Zettl, Evan J Reed, and Xiang Zhang. Structural phase transition in monolayer MoTe2 driven by electrostatic doping. *Nature*, 550:487, 2017.

[3] Carl H. Naylor, William M. Parkin, Jinglei Ping, Zhaoli Gao, Yu Ren Zhou, Youngkuk Kim, Frank Streller, Robert W. Carpick, Andrew M. Rappe, Marija Drndić, James M. Kikkawa, and A. T.Charlie Johnson. Monolayer single-crystal 1T-MoTe 2 grown by chemical vapor deposition exhibits weak antilocalization effect. *Nano Letters*, 16(7):4297–4304, 2016.

[4] Line Jelver, Peter Mahler Larsen, Daniele Stradi, Kurt Stokbro, and Karsten Wedel Jacobsen. Determination of low-strain interfaces via geometric matching. *Physical Review B*, 96 (8):085306, 2017.

[5] Daniele Stradi, Line Jelver, Søren Smidstrup, and Kurt Stokbro. Method for determining optimal supercell representation of interfaces. *Journal of Physics Condensed Matter*, 29 (18), 2017. ISSN 1361648X. doi: 10.1088/1361-648X/aa66f3.

[6] Junfeng Zhang, Weiyu Xie, Jijun Zhao, and Shengbai Zhang. Band alignment of two-dimensional lateral heterostructures. *2D Materials*, 4(1):15038, 2017. URL `http://stacks.iop.org/2053-1583/4/i=1/a=015038`.

[7] Ferney A Chaves and David Jiménez. Electrostatics of two-dimensional lateral junctions. *Nanotechnology*, 29(27):275203, 2018. ISSN 0957-4484. doi: 10.1088/1361-6528/aabeb2. URL `http://dx.doi.org/10.1088/1361-6528/aabeb2`.

[8] O. Leenaerts, S. Vercauteren, B. Schoeters, and B. Partoens. System-size dependent band alignment in lateral two-dimensional heterostructures. *2D Materials*, 3(2):025012, 2016.

[9] Henry Yu, Alex Kutana, and Boris I. Yakobson. Carrier Delocalization in Two-Dimensional Coplanar p-n Junctions of Graphene and Metal Dichalcogenides. *Nano Letters*, 16(8):5032–5036, 2016. doi: 10.1021/acs.nanolett.6b01822.

[10] Ankur Nipane, Sirisha Jayanti, Abhinandan Borah, and James T. Teherani. Electrostatics of lateral p-n junctions in atomically thin materials. *Journal of Applied Physics*, 122(19), 2017. doi: 10.1063/1.4994047.
